# Building Health Services in a Rapidly Changing Landscape: Lessons in Adaptive Leadership and Pivots in a COVID-19 Remote Monitoring Program

**DOI:** 10.2196/25507

**Published:** 2021-01-13

**Authors:** Celia Violet Laur, Payal Agarwal, Geetha Mukerji, Elaine Goulbourne, Hayley Baranek, Laura Pus, R Sacha Bhatia, Danielle Martin, Onil Bhattacharyya

**Affiliations:** 1 Women's College Hospital Institute for Health System Solutions and Virtual Care Toronto, ON Canada; 2 Department of Family and Community Medicine University of Toronto Toronto, ON Canada; 3 Department of Medicine, Division of Endocrinology & Metabolism University of Toronto Toronto, ON Canada; 4 Women's College Hospital Toronto, ON Canada; 5 University Health Network Toronto, ON Canada; 6 Dalla Lana School of Public Health University of Toronto Toronto, ON Canada

**Keywords:** adaptive leadership, pivots, acute care, COVID-19, leadership, remote monitoring, monitoring, health service, framework

## Abstract

Adaptive leadership has become an essential skill for leaders in health systems to respond to the COVID-19 pandemic as new knowledge emerges and case counts rise, fall, and rise again. This leadership approach has been described as an iterative process of taking a wide view of the situation, interpreting the meaning of incoming data from multiple directions, and taking real-time action. This process is also common in start-ups, which attempt to create new products or services of uncertain value for consumer markets that may not yet exist. Start-ups manage uncertainty through “pivots,” which can include changes in the target group, need, features, or intended benefit of a product or service. Pivots are large changes that account for the high likelihood of getting something wrong during development, and they are distinct from the “tweaks” or small tests of change that define quality improvement methodology. This case study describes three pivots in the launch of a remote monitoring program for COVID-19. Adaptive leadership helped inform strategic decisions, with pivots providing a framework for internal and external stakeholders to articulate options for changes to address shifting needs. There is considerable uncertainty in the appropriate design and implementation of health services, and although this case example focuses on the use of adaptive leadership and pivots during a pandemic, these strategies are relevant for health care leaders at any time.

## Introduction

Addressing the COVID-19 pandemic has required many shifts in strategy as new knowledge about the disease, its trajectory and spread, and its treatment has emerged. Health system leaders have been required to adapt health care delivery to clinical and system uncertainty as well as to the changing demographics of the populations that are most affected by the disease. For example, in mid-March 2020, the government of the Canadian province of Ontario encouraged the transfer of stable hospitalized patients to long-term care to prepare for a surge in hospital admissions due to COVID-19 [1]. By mid-April 2020, hospitals were mostly empty, so these transfers were paused; by mid-May, no patients were being transferred from hospitals to long-term care, and 82% of the deaths due to COVID-19 in Canada were occurring in long-term care facilities [2]. Protecting the capacity of acute care hospitals to prepare for the surge in cases led to strategic shifts toward, and then away from, transfers to long-term care in reaction to new information.

Adaptive challenges, or problems that cannot be solved by applying “current technical know-how or routine behavior,” can be managed using adaptive leadership, as first proposed by Heifetz in 1994 [3]. Key aspects of this leadership style include taking a wider view of the situation, interpreting what is really going on, and taking real-time action to rapidly ameliorate the situation in response to the perceived or projected needs [3]. In other words, adaptive leadership is about *anticipating* future needs, trends and options; *articulating*** **these needs to build collective understanding and support for action; *adapting* to allow continuous learning and the adjustment of responses as necessary; and having *accountability,* including transparency in decision-making processes and openness to challenges and feedback [4]. These skills are all essential during a pandemic [3,5-8].

The level of uncertainty in health care has risen substantially during the COVID-19 pandemic. Practical approaches to managing extreme uncertainty may come from groups that are accustomed to this management, such as the founders of start-up companies. Start-ups are highly practiced in adaptive leadership because they develop products and services that do not yet exist and may not be effective for a consumer market that may not materialize. To address this challenge, start-ups have operationalized many adaptive strategies, most notably the concept of “pivots,” which are used to match a service to a public need [9]. These include large changes to programs, such as narrowing or expanding the set of functions, changing customer segments, focusing on a different customer need, or changing delivery channels [9]. For example, the makers of Burbn, an app that allowed users to check in, post their plans, and share photos, noted that use of the first two functions was limited; therefore, they “zoomed in” on the photo-sharing feature and relaunched as Instagram [10]. YouTube began as a video dating website and successfully “zoomed out” to become the video streaming service known today [11]. When applied to health care, pivots may help organizations articulate different options that can be tested quickly to meet health system needs during times of extreme clinical and system uncertainty.

Pivots and adaptive leadership appear to be complementary. Adaptive leadership supports the program team in making required changes in values, beliefs, and behaviors so they can continue to meet the needs of their patients and of the health care system. Ideally, this approach helps teams to create meaning for large changes, potentially protecting against change fatigue, and positions the management of constant change as a core skill for the teams. Pivots provide a framework to facilitate difficult strategic decisions.

**Figure 1:**
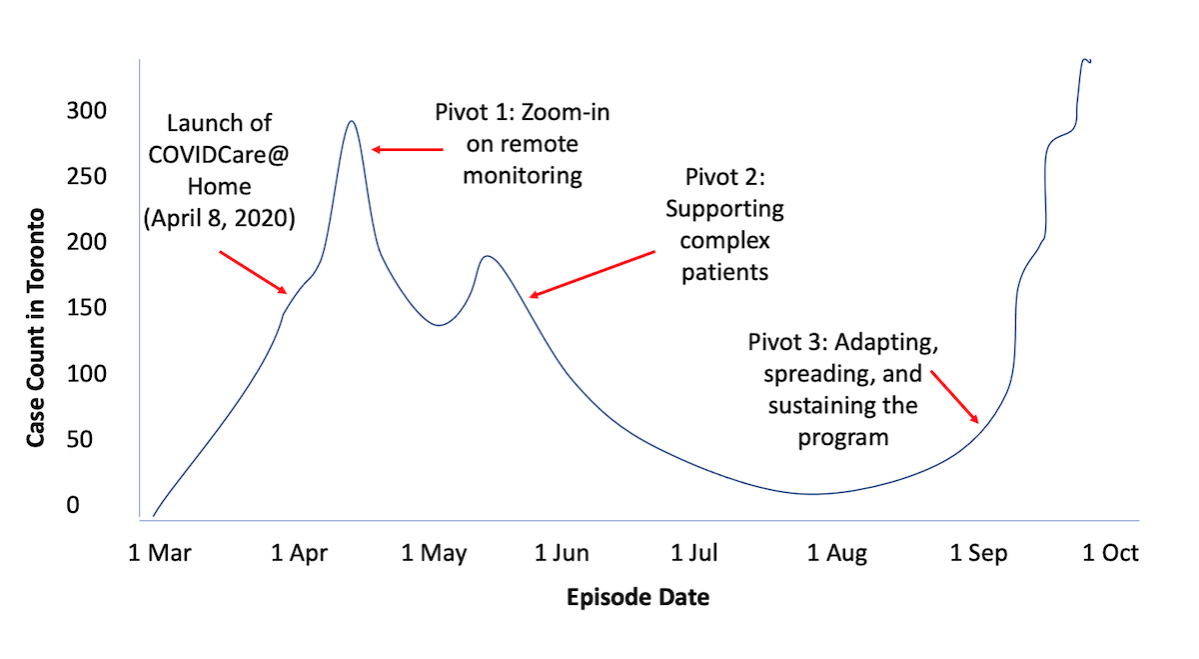
Mapping the pivots of the COVIDCare@Home program to an approximation of the number of cases (confirmed and probable) of COVID-19 in Toronto, Ontario, Canada, from March to October 2020.

**Figure 2:**
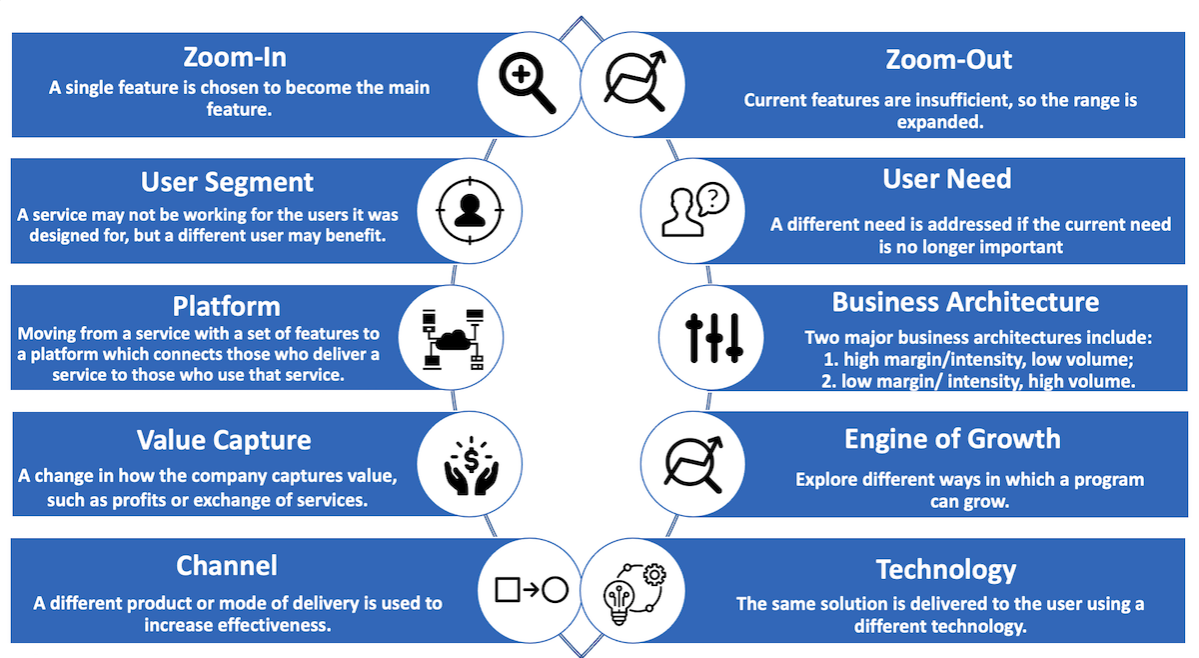
Names and brief descriptions of pivots. Images were obtained from the Noun Project [15].

In March 2020, as the number of people testing positive for COVID-19 in Canada was rising [12], Women’s College Hospital, an academic ambulatory hospital in downtown Toronto, a diverse city with a population of approximately 3 million, saw a need to support people in the community who tested positive for COVID-19. This need led to the development of COVIDCare@Home (CC@H), a remote monitoring program to support people who test or are presumed to be positive for COVID-19 in their homes. CC@H offers remote monitoring using telephone and video visits 7 days a week by an interprofessional, family medicine–led team, which aimed to follow patients during the acute phase of the illness (typically 14 days from symptom onset) or until they were discharged to community-based care from their primary care provider. To accelerate the process, senior hospital leaders enabled program leads to make rapid, informed, and strategic choices, drawing on principles of user-centered design with patient and provider interview feedback collected during the initial development [13,14]. This case study describes the application of adaptive leadership and three of the key pivots that allowed CC@H to launch within one week and adapt to the changing needs of patients with COVID-19 during a period of rapid changes in clinical and health system needs. A timeline of the pivots in relation to COVID-19 case rates in Toronto is included in Figure 1. Descriptions of the named pivots are visualized in Figure 2, and detailed examples based on CC@H are provided in Table 1.

**Table 1:** Names, descriptions, and examples of the pivots that are mainly relevant to COVIDCare@Home. The pivots were named by Ries [9] and adapted for the health care context by the authors. “User” can refer to patients, customers, or other groups.

Pivot category and name		Description		Example	
**Pivots applied to CC@H**					
	Zoom-in		A single feature is chosen to become the main feature and everything else is cut away, thus optimizing delivery of this feature and its value proposition.		The CC@H team quickly “zoomed in” to focus on remote monitoring of community-based patients, while other strategies were deprioritized.
	Business architecture		The two major business architectures include (1) high margin/intensity, low volume and (2) low margin/intensity, high volume. These approaches cannot be applied simultaneously.		The CC@H program initially provided low-intensity care to a high volume of patients because the team initially predicted high demand, then pivoted to low volume/high intensity when the COVID-19 case counts decreased and the patients were found to be more medically and socially complex.
	Engine of growth		There are different ways in which a program can grow, such as changing the cost structure to make better use of existing resources; encouraging policy changes to generate new revenue sources; or developing a hub-and-spoke model to support replication in other sites.		The second wave of COVID-19 required CC@H to move from a short-term program with high resource use to a long-term sustainable program with limited resources, such as by decreasing reliance on the use of high-cost staff such as physicians.
	Channel		Changing to a different product or mode of delivery to increase effectiveness.		Many health care institutions changed their primary channel of delivery during the COVID-19 pandemic when moving from in-person to virtual patient visits. Another example is the movement of CC@H from primarily using video visits with patients to using digital surveys to triage patients.
	Technology		The same solution is delivered to the user using a completely different technology, such as when new technology is available at better value.		The CC@H team began sending pulse oximeters and thermometers to support monitoring rather than only using paper-based systems for tracking physiological parameters.
**Pivots that *could* be applied to CC@H**					
	Zoom-out		The reverse of the zoom-in pivot. When the current features are insufficient for the user, the range of features is expanded.		To support the higher-intensity approach, CC@H could expand their services, such as by providing home care in addition to remote monitoring for patients at higher risk in the community.
	User segment		A service may not be interesting to the users it was designed for, but early insights suggest a different user may benefit.		In the future, CC@H could change their patient population, such as to focus on postdischarge or complex patients who may receive more benefit from this approach.
	User need		If early user feedback shows that the problem being solved is not very important, the team may pivot to address a different need.		As evidence of post-acute COVID-19, also known as “long COVID,” emerges [16] and emphasis on new cases decreases, CC@H may move away from focusing only on supporting recently diagnosed patients to include longer follow-up of patients with continuing symptoms.
	Platform		A service with a set of features changes to a platform that connects those who deliver a service to those who use that service.		CC@H could shift to a remote monitoring platform that connects specialty services to a multidisciplinary team to remotely monitor patients with different conditions.
	Value capture		The is a monetization or revenue model. Leaders change how the company captures value, such as increased focus on profits or exchange of services.		CC@H could change to offering a paid service to other institutions or include it as part of inpatient care for hospital partners to allow for reimbursement in a bundled payment.

^a^CC@H: COVIDCare@Home

## Pivot 1: Zooming In on Remote Monitoring

In Ontario, modeling data presented on April 3, 2020, projected that the demand for intensive care unit beds would exceed the capacity by mid-April, with a projected 80,000 cases by April 30 if current measures were followed [17]. There were concerns that hospitals would become overwhelmed. The literature contained one well-described model for remote monitoring in primary care [18]. In a desire to keep people out of hospital who could safely care for themselves at home with support, CC@H was launched by Women’s College Hospital on April 8, 2020. Partners in this program included the Department of Family and Community Medicine at the University of Toronto and Mount Sinai Hospital, an acute care academic hospital that is part of the Sinai Health System.

Initial strategies of CC@H included setting up a telephone line to provide primary care providers with access to expertise in social work, pharmacy, nurse navigation, general internal medicine, respirology, and psychiatry. The team also developed resources to support early discharge from acute care and developed a protocol for remote monitoring of patients in the community [19]. CC@H quickly “zoomed in” (Table 1) on remote monitoring of community-based patients within the Greater Toronto Area who tested positive for COVID-19, while other strategies were deprioritized. This zoom-in pivot enabled the team to focus resources on refining remote monitoring processes, including video and telephone visits, methods for remote triaging, clinical pathways to address symptoms, and use of devices, such as sending pulse oximeters and thermometers to patients. Remote monitoring services followed 2020 recommendations from Greenhalgh et al [18] and were made available 24 hours per day for up to 14 days. Details are published elsewhere [20].

For initial staffing*,* a primary care, team-based approach was used, relying on redeployed physicians and staff from Women’s College Hospital, primary care residents, and a multidisciplinary team (MDT) of providers. The MDT included nurses, a pharmacist, social workers, mental health workers, and other available specialists, who worked together to remotely address clinical needs as well as the social determinants of health of the patients.

## Pivot 2: Supporting Complex Patients

The original aim of CC@H aligned with system projections that support would be needed for a high volume of patients who tested positive for COVID-19 at a low intensity, including occasional contact with mechanisms for escalation as needed [17]. However, with the initial health system focus on protecting acute care [1] and then on long-term care [2], there was delayed recognition of the need to support underserved populations who were at high risk, such as those in congregate living settings or whose low incomes, precarious work, or housing situations made their social situations particularly complex [21-23]. For CC@H, this meant that fewer patients were admitted to the program than anticipated; however, those who were admitted had complex needs beyond their COVID-19 diagnosis [20].

By early May, half of the patients admitted to CC@H had one or more comorbidity [20], and 56% belonged to occupational groups who are more likely to contract COVID-19 (such as personal support workers, shelter workers, and cleaners) and to have social challenges (such as food insecurity or lack of access to financial support), which increased the risk of poor health outcomes [20,21]. For these reasons, the program pivoted from high patient volume with low patient contact to low patient volume with high service intensity. There was an average of 4.4 visits per patient in the first month [20], with visits focused on monitoring symptoms and addressing medical and social needs, and subsequent visits were scheduled at the end of each visit based on patient preferences and clinical judgment. This type of shift in intensity is called a business architecture pivot (Table 1), which posits that a business can be low-margin, high-volume or high-margin, low-volume but not both [9]. The analogous situation in health care is a shift in the intensity and volume of a service, which in this case allowed for a more patient-centered approach that supported complex patients. The pivot facilitated use of services such as access to mental health support (ie, brief counseling and access to community resources), navigation of government support (ie, providing information on what financial or other programs the patient was eligible for and how to apply), and strategies to address food insecurity (ie, providing information on grocery delivery services, food banks, and other local initiatives available during the pandemic).

Regarding staffing changes*,* as the program grew, the original plan was to recruit more physicians and registered nurses (RNs) to support the high volume of patients with COVID-19–specific needs. However, with the focus on supporting patients with complex needs, a nurse practitioner (NP) was assigned instead. The NP could focus on case management specifically for complex patients who needed more intense support and delivered clinical care when the number of patients in the program increased. The emphasis was on comprehensive care, and social workers and mental health professionals also became more involved in case management.

## Pivot 3: Adapting, Spreading and Sustaining the Program

When the number of COVID-19 cases decreased across Ontario in July and August 2020 [24], CC@H responded by ramping down; redeployed staff were allowed to return to their original roles, and many participating primary care physicians returned to their prepandemic practice models. The program remained nimble, retaining the ability to service higher volumes if needed. In September, when a second wave of cases began to build with predominately younger and lower-risk patients [24], a new emphasis was placed on improving triage. This approach included light-touch digital monitoring systems, such as electronic survey questions for low-risk patients, to help triage patient risk and increase the frequency of monitoring.

This second wave of COVID-19 cases necessitated an engine of growth pivot (Table 1), moving from a short-term program with high resource use and access to redeployed staff to a long-term sustainable program with limited resources and a staffing model that did not rely on redeployment. To achieve this change, the program leaders decreased reliance on the use of high-cost staff such as physicians. With increasing clinical confidence, the program team felt more comfortable with minimal physician contact for low-risk patients, enabling physicians to prioritize complex and high-risk patients. “Digital Care Coach,” an electronic medical record (EMR) tool that includes a symptom questionnaire to enable low-touch remote monitoring, was prepared, and triaging guidelines (criteria for low-, medium-, and high-risk patients based on symptoms, patient history, and clinical judgment) were adapted to enable longer times between virtual visits. This EMR tool monitored symptoms of low-risk patients and provided educational materials, allowing the program to provide care to more patients while maintaining staffing levels and helping providers to focus on higher-risk patients.

Regarding staffing changes and communication strategies, to run a more sustainable program, the number of physicians involved in the program did not increase in proportion to the number of patients; instead, the program relied on more NPs and RNs to manage lower-risk patients, supported by digital tools such as Digital Care Coach. Communication strategies also became more sustainable, with the use of a patient roster and weekly clinical case conferences rather than daily huddles. The emphasis shifted to effective use of time and resources for a longer-term program.

## Barriers and Enablers

Significant enablers of the rapid launch of CC@H included the suspension of many elective activities in the hospital as well as a sense of urgency. The leadership commitment to rapid action helped overcome the typical barriers and delays associated with building new programs in large organizations. Senior leaders worked closely with the program lead to ensure the necessary staffing, resources, and information technology support were available. The program lead facilitated integration of the EMR system into the program workflow, responded to stakeholder feedback in real time, developed more efficient processes for care delivery, and thus built trust among the team that enabled future pivots. Everyone involved saw the need for this program and worked through several hurdles to meet this need. Among those hurdles was adapting the EMR system to meet the changing needs of the program, which led to a steep learning curve for providers who were accustomed to a different EMR.

Maintaining the appropriate staffing levels was also challenging due to the fluctuating case numbers. A core group of physicians were involved for several months at a time, varying their number of hours per week with the program, rather than adding new providers. The social work and mental health professionals and pharmacists remained constant throughout; however, there was high turnover among RNs and NPs. Providers were flexible, moving between several programs across Women’s College Hospital based on program needs and provider skills. Providers were aware of this shifting need, and training was provided to facilitate transitions. With increasing focus on the social determinants of health, more mental health and social workers were needed, and the team was required to keep up to date on the services that were still open to know which service to recommend.

The rapid speed at which pivots occurred was both an enabler and a barrier. Changes were enacted quickly (typically within a few days) to meet the needs of patients, which limited the time for team consultation and led to a more top-down approach. The daily huddles and weekly meetings enabled the team to be informed of changes quickly and to be involved in ensuring that the changes worked for them. Although decisions to pivot were made by leadership, those decisions were informed by the team and adapted based on their continuous input in the huddles.

The decision to make this program primary care–led was another significant enabler, as it allowed for a holistic approach to care, addressing issues beyond the COVID-19 diagnosis, including social determinants of health. The primary care approach may help with the spread of the program, as it can be used in low-resource settings and in any primary care–led facility, such as long-term care. The program could also be adapted for patients who do not have COVID-19 and can be integrated into the general delivery of primary care. This remote monitoring approach did have several barriers, particularly regarding the use of technology, as many patients were unable to access video visits due to device compatibility, internet bandwidth, and other reasons. The team had to rely on different methods, including providing care via telephone or other video calling methods, such as FaceTime.

System-level enablers included increased access to human resources for pandemic-specific programs. The launch of medical billing codes for telephone and video visits facilitated physician involvement though financial renumeration of virtual care services provided. The availability of medical residents whose rotations were suspended was key, as the residents were able to serve in the model and quickly adapted to new systems and ways of working. System-level barriers included the limitations of billing codes to support case conferences and coordination.

## Discussion

The rapid launch and strategic pivots enacted through adaptive leadership in CC@H enabled the team to continue to meet the needs of their patients through different waves of the pandemic. This provides a concrete example of how adaptive leadership in health care can support important outcomes in times of uncertainty [5-7].

There is considerable uncertainty in the appropriate design and implementation of health services, and although this case example focuses on the use of adaptive leadership and pivots at the organization level during a pandemic, these strategies are relevant for health care leaders at any time. These leadership skills can be learned, and the use of specified pivots may help describe options for major modifications based on emerging evidence and facilitate this adaptive approach in practice. This case focuses on individual- and organizational-level changes, and further work is needed to consider applicability at the population level, such as how elected leaders may use this approach and how to prepare the population for these rapid changes. Using this approach takes practice and courage. While the literature provides basic steps [3], there are few documented examples of the types of choices that are involved in practicing this form of leadership.

Training in the design and deployment of new services can facilitate this leadership strategy, as can the use of real-time data in implementation and outcome measures to guide decisions. Training for all staff on this adaptive approach would also be beneficial so the team members can be ready for rapid change, understand their role in the change process, and know when and how to provide feedback. This rapid feedback process will be important for staff and leadership to ensure that the pivot aligns with the needs of the team and that they have the trust of the team to go forward. The “balcony view” clearly articulated in adaptive leadership theory also acknowledges the need for system-level thinking to meet the needs of the health care system, providers, and patients. The combination of adaptive leadership and pivots provides a mechanism for making major changes in a complex system such as health care.

Additional contextual factors may have enabled the use of adaptive leadership in CC@H, such as initial close communication with senior leadership, access to staff from different disciplines, and a dedicated program lead who had training in systems engineering and had worked at start-ups. Although some of these factors are difficult to reproduce or sustain, clear communication from senior leadership and trust among team members are key and are achievable in other settings to facilitate use of adaptive leadership.

Comparison to other COVID-19 remote monitoring programs highlights directions that could have been taken by CC@H. For example, a model from Minneapolis used newsfeeds with reminders and daily check-in questionnaires about symptoms. Initially, CC@H started to develop a similar dashboard; however, while the Minneapolis program was monitoring over 1300 patients [10], CC@H only had 100 patients in the first month. Without the change in intensity (Pivot 2), CC@H would have continued to provide light-touch care for patients and would not have been able to provide the comprehensive care and case management needed for patients with complex needs at the time. However, in the second wave (Pivot 3), use of dashboards and low-touch monitoring became a priority to make the program more sustainable given the rising number of low-risk patients.

## Conclusion

The CC@H program is using adaptive leadership and pivots to nimbly adapt to meet the changing needs of their patients during this time of clinical and system uncertainty, demonstrating the value of this approach. By using pivots as a framework for large strategic changes rather than small tweaks, pivots can provide direction and meaning to support health system leaders as they quickly adapt to changing needs in health care. This combination of adaptive leadership and pivots is broadly relevant at most levels of health care leadership.

## Abbreviations

CC@H: COVIDCare@Home
EMR: electronic medical record
NP: nurse practitioner
RN: registered nurse
