# Correction: Building Health Services in a Rapidly Changing Landscape: Lessons in Adaptive Leadership and Pivots in a COVID-19 Remote Monitoring Program

**DOI:** 10.2196/31044

**Published:** 2021-06-10

**Authors:** Celia Violet Laur, Payal Agarwal, Geetha Mukerji, Elaine Goulbourne, Hayley Baranek, Laura Pus, R Sacha Bhatia, Danielle Martin, Onil Bhattacharyya

**Affiliations:** 1 Women's College Hospital Institute for Health System Solutions and Virtual Care Toronto, ON Canada; 2 Department of Family and Community Medicine University of Toronto Toronto, ON Canada; 3 Department of Medicine, Division of Endocrinology & Metabolism University of Toronto Toronto, ON Canada; 4 Women's College Hospital Toronto, ON Canada; 5 University Health Network Toronto, ON Canada; 6 Dalla Lana School of Public Health University of Toronto Toronto, ON Canada

In “Building Health Services in a Rapidly Changing Landscape: Lessons in Adaptive Leadership and Pivots in a COVID-19 Remote Monitoring Program” (J Med Internet Res 2021;23(1):e25507), one error was noted.

In the originally published article, author Danielle Martin was associated with the incorrect ORCID number. In the corrected article, the ORCID number for this author has been updated as follows:

0000-0003-2517-2602

The correction will appear in the online version of the paper on the JMIR Publications website on June 10, 2021, together with the publication of this correction notice. Because this was made after submission to PubMed, PubMed Central, and other full-text repositories, the corrected article has also been resubmitted to those repositories.

